# Risk factors for long coronavirus disease 2019 (long COVID) among healthcare personnel, Brazil, 2020–2022

**DOI:** 10.1017/ice.2023.95

**Published:** 2023-12

**Authors:** Alexandre R. Marra, Vanderson Souza Sampaio, Mina Cintho Ozahata, Rafael Lopes, Anderson F. Brito, Marcelo Bragatte, Jorge Kalil, João Luiz Miraglia, Daniel Tavares Malheiro, Yang Guozhang, Vanessa Damazio Teich, Elivane da Silva Victor, João Renato Rebello Pinho, Adriana Cypriano, Laura Wanderly Vieira, Miria Polonio, Solange Miranda de Oliveira, Victória Catharina Volpe Ricardo, Aline Miho Maezato, Gustavo Yano Callado, Guilherme de Paula Pinto Schettino, Ketti Gleyzer de Oliveira, Rúbia Anita Ferraz Santana, Fernanda de Mello Malta, Deyvid Amgarten, Ana Laura Boechat, Takaaki Kobayashi, Eli Perencevich, Michael B. Edmond, Luiz Vicente Rizzo

**Affiliations:** 1 Hospital Israelita Albert Einstein, São Paulo, São Paulo, Brazil; 2 Department of Internal Medicine, University of Iowa, Iowa City, Iowa, United States; 3 Instituto Todos Pela Saúde, São Paulo, São Paulo, Brazil; 4 West Virginia University School of Medicine, Morgantown, West Virginia, United States

## Abstract

**Objective:**

To determine risk factors for the development of long coronavirus disease 2019 (COVID-19) in healthcare personnel (HCP).

**Methods:**

We conducted a case–control study among HCP who had confirmed symptomatic COVID-19 working in a Brazilian healthcare system between March 1, 2020, and July 15, 2022. Cases were defined as those having long COVID according to the Centers for Disease Control and Prevention definition. Controls were defined as HCP who had documented COVID-19 but did not develop long COVID. Multiple logistic regression was used to assess the association between exposure variables and long COVID during 180 days of follow-up.

**Results:**

Of 7,051 HCP diagnosed with COVID-19, 1,933 (27.4%) who developed long COVID were compared to 5,118 (72.6%) who did not. The majority of those with long COVID (51.8%) had 3 or more symptoms. Factors associated with the development of long COVID were female sex (OR, 1.21; 95% CI, 1.05–1.39), age (OR, 1.01; 95% CI, 1.00–1.02), and 2 or more SARS-CoV-2 infections (OR, 1.27; 95% CI, 1.07–1.50). Those infected with the SARS-CoV-2 δ (delta) variant (OR, 0.30; 95% CI, 0.17–0.50) or the SARS-CoV-2 o (omicron) variant (OR, 0.49; 95% CI, 0.30–0.78), and those receiving 4 COVID-19 vaccine doses prior to infection (OR, 0.05; 95% CI, 0.01–0.19) were significantly less likely to develop long COVID.

**Conclusions:**

Long COVID can be prevalent among HCP. Acquiring >1 SARS-CoV-2 infection was a major risk factor for long COVID, while maintenance of immunity via vaccination was highly protective.

Most people infected by the SARS-CoV-2 virus tend to have complete resolution of their symptoms within a few days to a few weeks after infection, but some develop prolonged symptoms following acute infection.^1,2^ The post-coronavirus disease (COVID) conditions, also known as long COVID, according to the Centers for Disease Control and Prevention (CDC), include a wide range of new, returning, or ongoing health problems that people experience 4 or more weeks after first being infected with the virus that causes COVID-19.^1^ It has been estimated that ∼200 million individuals have experienced these prolonged symptoms, with a global prevalence of 43% of the infected.^2^

In the third year of the COVID-19 pandemic, 68.3% of the world population received at least 1 dose of a COVID-19 vaccine. It has already been proven that in fully vaccinated populations, the vaccine effectiveness was high against infection (89.1%) and severe outcomes such as hospitalization (97.2%), admission to an intensive care unit (97.4%), and death (99.0%).^3^ However, despite the high effectiveness of the available vaccines,^4–6^ parts of the population remain susceptible to infection and long COVID-19.^7–9^ Moreover, long COVID has been linked to >3,500 deaths emphasizing the need for the prevention of infection.^9^

Studies have shown that long COVID can follow both mild acute disease and the most severe forms.^10–12^ Risk factors for long COVID in nonhospitalized adults include female sex, socioeconomic deprivation, obesity, and a wide range of comorbidities.^12^ Healthcare personnel (HCP) have been identified as more vulnerable to infection due to their high frequency of occupational exposure.^13,14^ Recent studies have shown that most vaccinated individuals report improvement in long COVID symptoms.^15^ Among the few studies available, a cohort study that followed HCP found that the number of vaccine doses was associated with a lower prevalence of long COVID.^16^ Additional studies are needed to evaluate the effectiveness of COVID-19 vaccines against long COVID among HCP.^17–19^ In the present study, we analyzed epidemiological data and risk factors for the development of long COVID among HCP in Brazil.

## Methods

### Population and setting

This case–control study included HCP (aged ≥18 years) at Hospital Israelita Albert Einstein (HIAE) from March 1, 2020, to July 15, 2022. The HIAE is a Brazilian nonprofit healthcare, educational, and research organization, headquartered in the city of São Paulo, that manages a diverse healthcare system ranging from primary healthcare to tertiary-care services in the public and private healthcare sectors. It operates 40 healthcare units, mainly in the state of São Paulo, and in 2021 it had ∼870,000 emergency department visits, ∼1,000,000 outpatient visits, and ∼87,000 hospital discharges. Since the beginning of the COVID-19 pandemic, HCP with COVID-19 symptoms had access to free-of-charge SARS-CoV-2 reverse-transcriptase polymerase chain reaction (RT-PCR) testing conducted by the institution’s laboratory. During the study period, the Oxford-AstraZeneca [ChAdOx1], CoronaVac, Pfizer/BioNTech, and Janssen vaccines were available at our hospital (Supplementary Appendix 1). HCP were required to notify the employee health clinic if they tested positive for SARS-CoV-2. HCP with a laboratory-confirmed COVID-19 by reverse transcriptase polymerase chain reaction (RT-PCR) were assessed by the employee health clinic at 1, 3, and 6 months after the first infection via in-person appointment, telephone appointment, or emails.

We included HCP with a laboratory-confirmed COVID-19 by RT-PCR. RT-PCR testing for the diagnosis of COVID-19 was only performed on symptomatic HCP. Cases were classified as those developing long COVID, defined as signs and symptoms that developed during or following a SARS-CoV-2 RT-PCR confirmed infection, continued for >4 weeks, and could not explained by an alternative diagnosis.^1^ The symptoms considered related to long COVID were general symptoms (eg, fever, tiredness or fatigue), respiratory and heart symptoms (eg, shortness of breath, cough, chest pain, heart palpitation), neurological symptoms (eg, headache, difficulty concentrating, change in smell or taste, depression or anxiety), digestive symptoms (eg, diarrhea, stomach pain), or other symptoms (eg, joint or muscle pain).^1^ HCP who recovered from acute COVID-19 within 4 weeks were classified as controls (Supplementary Appendix 2 and 3).

### Real-time polymerase chain reaction (RT-PCR) methodologies for SARS-CoV-2 detection

Diagnostic confirmation for COVID-19 was performed using RT-PCR on specimens obtained via nasopharyngeal swab, according to the protocol instituted at HIAE. The following RT-PCR kits were utilized: XGEN MASTER COVID-19 (Mobius, Pinhais, Paraná, Brazil), cobas SARS-CoV-2 Test (Roche Molecular Systems, Branchburg, NJ), Xpert Xpress SARS-CoV-2 (Cepheid, Sunnyvale, CA), and Abbott RealTime SARS-CoV-2 (Abbott Molecular, Des Plaines, IL).

### Next-generation sequencing of the viral full-length genome

We extracted total nucleic acid from naso-oropharyngeal (NOP) swab samples with the QIAamp Viral RNA Mini kit (QIAGEN, Hilden, Germany). After purification and concentration, DNAse I treatment, and depletion of human ribosomal RNA, samples were submitted to random amplification.^20^ Preparation of sequencing libraries for the Illumina platform was carried out with DNA Prep (Illumina, San Diego, CA) using the random 2-step PCR amplification product as input. Libraries were quantified with the Qubit instrument (Thermo Fisher Scientific, Waltham, MA) and loaded on the NextSeq 550 equipment (Illumina) for sequencing with MID 300 paired-end reads (Illumina).

### Exposures of interest and statistical analyses

Exposure variables were compared between those with and without long COVID to identify factors associated with development of long COVID. Demographic and clinical information of HCP, vaccination status, SARS-CoV-2 RT-PCR results, and presence or absence of long COVID were obtained from institutional electronic records including electronic medical records, laboratory reports from the microbiology laboratory, and separate records at the employee health clinic. Baseline exposure variables included sex, age, body mass index (BMI), physical activity (> or <30 minutes per day), self-reported hypertension, diabetes mellitus, arthritis, stroke, chronic kidney disease or cancer, job type (no direct patient contact or direct patient facing), the number of COVID-19 vaccine doses received prior to infection, homologous or heterologous (a booster dose different from the primary vaccine doses) COVID-19 vaccine scheme, the number of SARS-CoV-2 infections, and SARS-CoV-2 variant. HCP at our hospital require to be seen annually at the employee health clinic using standardized questionnaires assessing the presence of direct patient care, physical activity, etc. HCP were considered unvaccinated if no COVID-19 vaccine doses were received. We considered that HCP had reinfection when HCP has another positive RT-PCR testing 90 days or more from the first infection.^21^

**Figure 1.**
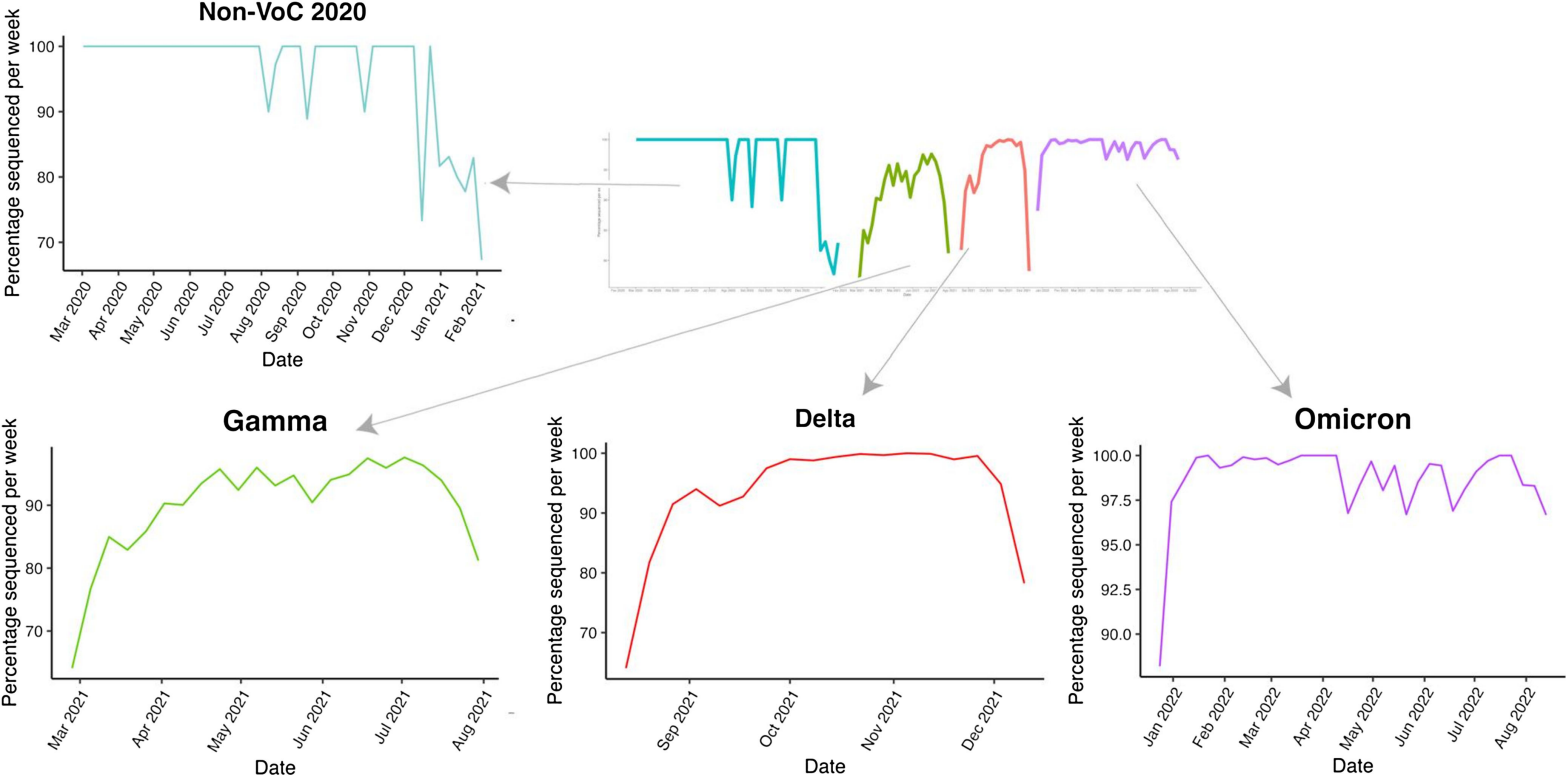
SARS-CoV-2 circulating variants. The figure shows the percentage of SARS-CoV-2 circulating variants sequences submitted to the Global Initiative on Sharing Avian Influenzae Data (GISAID) in São Paulo, Brazil, during the study period. Only variants with the most significant percentage are shown for each period. Individual plots for each variant era are shown at the top left and bottom of the figure.

Regarding the SARS-CoV-2 variants, because only a small number of positive samples among our HCP were sequenced, all individuals were classified in accordance to the most prevalent variant (≥75% of sequenced viruses) registered by the Global Initiative on Sharing Avian Influenza Data (GISAID) weekly in São Paulo, Brazil, at the time of the sample collection.^22^ In summary, the period between March 2, 2020, and February 11, 2021 was considered “non–variant-of-concern 2020 (non-VoC 2020) era”; February 26, 2021, to August 5, 2021, was considered the “gamma era”; August 13, 2021, to December 16, 2021, was considered the “delta era”; and December 24, 2021, to July 15, 2022, was considered the “omicron era” (Fig. 1).

The cumulative incidence of long COVID and its 95% confidence interval (CI) was estimated for the cohort. The univariate association between exposure variables and long COVID-19 was assessed using the χ^2^ or Fisher exact test, as appropriate. Additionally, the interaction between COVID-19 vaccine doses prior to infection, number of COVID-19 infections, and variant era as well as their association with long COVID were also tested. Multiple logistic regression models were developed to assess the independent association between exposure variables and long COVID. Odds ratios (ORs) and their respective 95% confidence intervals (CIs) were calculated. Exposure variables statistically significant in the simple models were included in the multiple logistic regression models. The signs and symptoms were counted, and the relative frequencies were used to estimate correlation and build the correlation matrix. R version 4.2.1 software with R Studio version 2022.07.1 (R Foundation for Statistical Computing, Vienna, Austria) was used for the analysis. All reported tests were 2-sided, and *P* < .05 was considered significant. The study was approved by the Hospital Israelita Albert Einstein Ethics Committee (CAAE 47110421.7.0000.0071), and the need for informed consent was waived.

## Results

**Figure 2.**
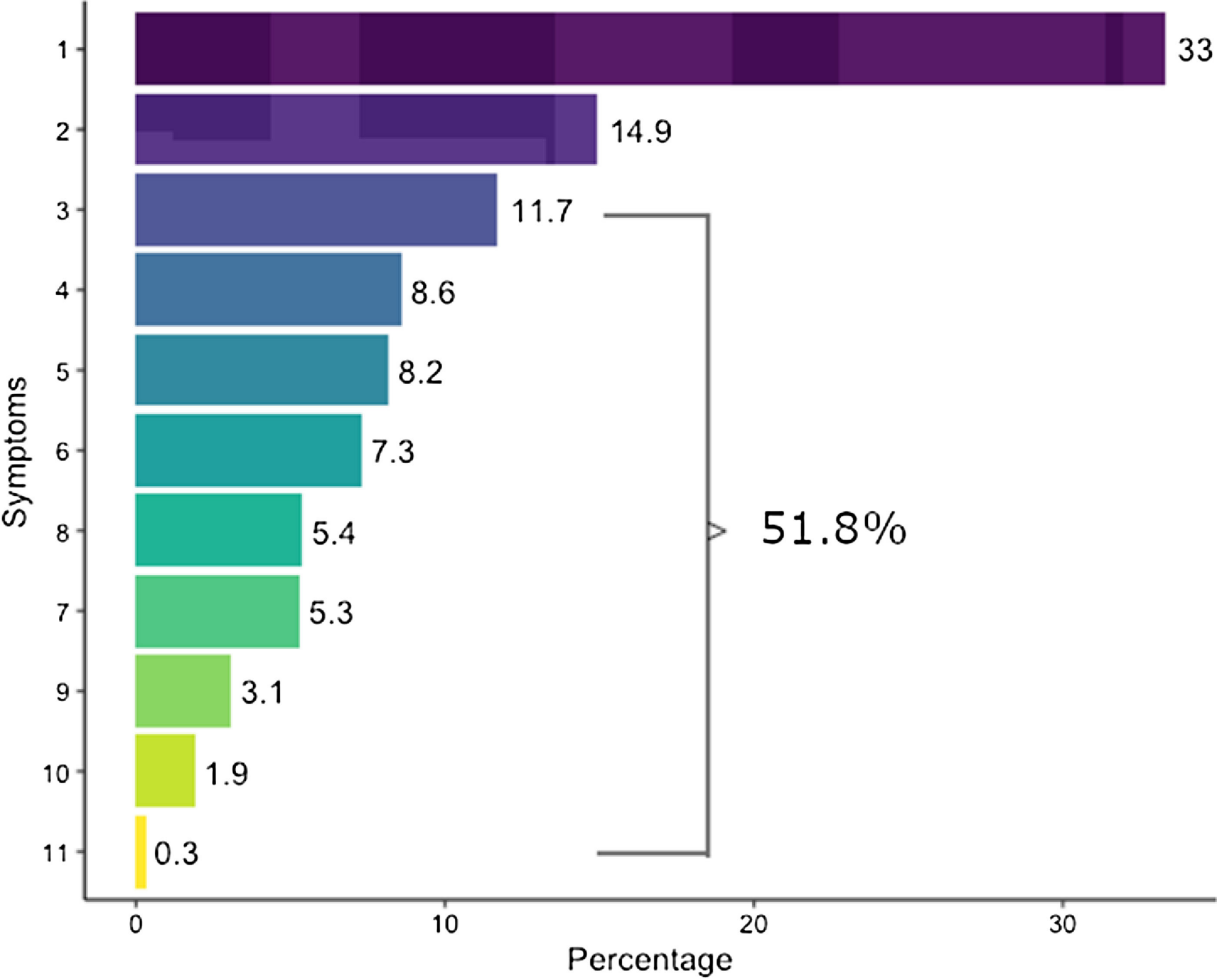
Number of long COVID-19 signs and symptoms per HCP.

**Figure 3.**
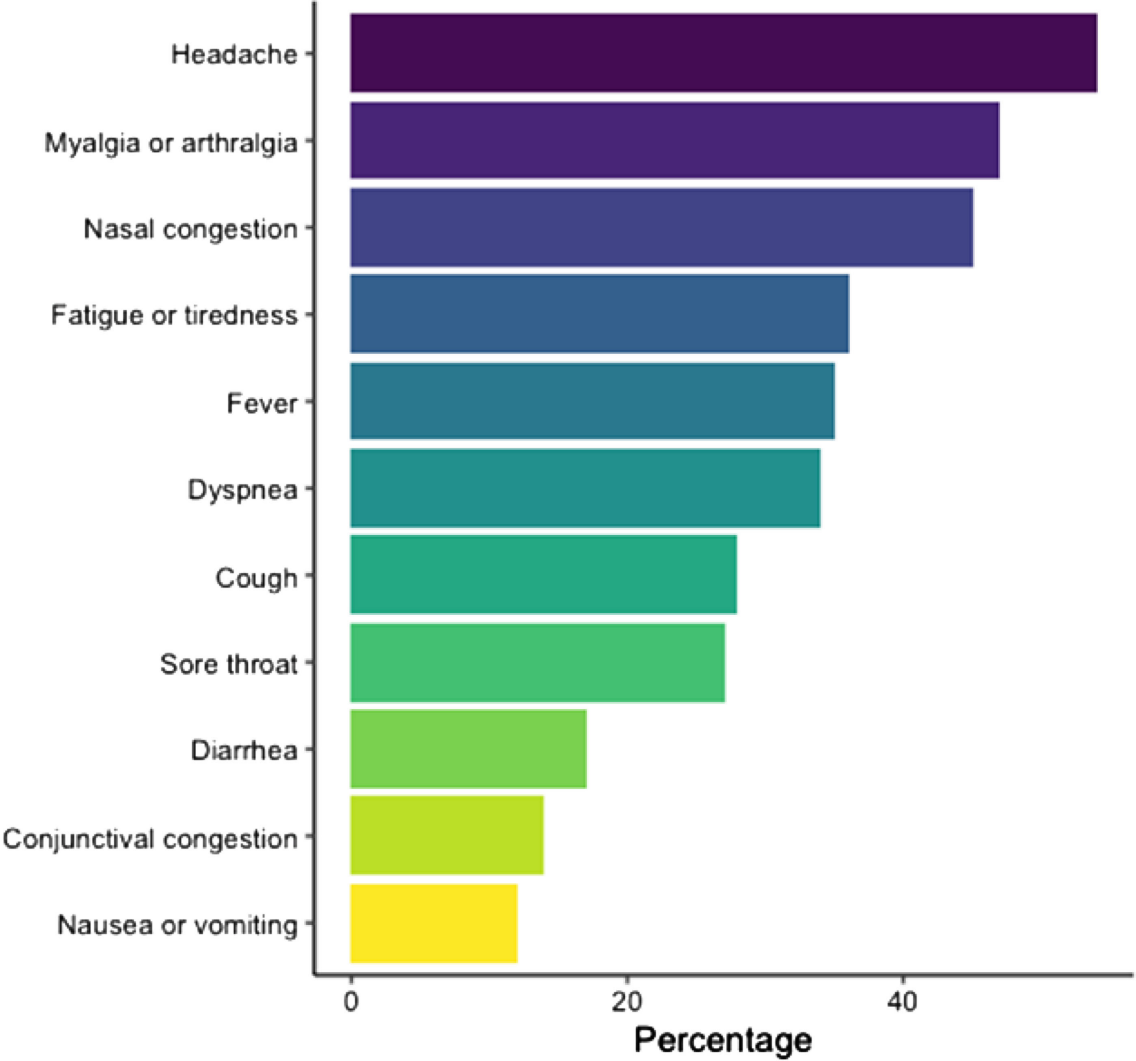
Most frequent symptoms of long COVID during 180 days of follow-up.

Among 18,340 total HCP at our institution, 7,051 HCP (38.4%) had at least 1 laboratory-confirmed SARS-CoV-2 infection during the study period. Of those infected, 1,933 HCP (27.4%) who developed long COVID were compared to the 5,118 (72.6%) who did not (ie, controls, Supplementary Appendix). Most of those with long COVID had 3 or more signs or symptoms (51.8%), whereas 644 had 1 infection (33.3%) and 288 (14.9%) had 2 infections (Fig. 2). The most common symptoms were headache (53.4%), followed by myalgia or arthralgia (46.6%), and nasal congestion (45.1%) (Fig. 3 and Supplementary Appendix 2).

### Unadjusted analysis

Of 7,051 HCP who had at least 1 SARS-CoV-2 infection, 5,101 were female (72.3%). Females represented 74.1% of the cases and 71.7% of the controls (*P* = .048). The mean age for the entire infected cohort was 37.5 years, 38.1 years for those with long COVID, and 37.2 years for those without long COVID-19 (*P* < .001). Mean BMI was 27.0 overall, 27.4 for those with long COVID, and 26.8 for those without long COVID-19 (*P* < .001). Information regarding comorbidities was available for 5,722 HCP (81.1%) in the infected cohort; at least 1 comorbidity was present in 28.9% overall: 31.1% in cases and 28.0% in controls (*P* = .021). The most prevalent comorbidities were hypertension (9.7% in cases and 9.0% in controls) and diabetes mellitus (3.5% in cases and 2.7% in controls) (Table 1). No difference was observed in physical activity between those with and without long COVID-19 (*P* = .472), and no difference was detected by the HCP job type (directly patient facing or not, *P* = .08). Of the infected HCP, 3,853 (54.6%) were vaccinated before COVID-19; 39.2% of cases were vaccinated (at least 1 dose) versus 60.5% of controls. A difference was observed in the number of vaccine doses before infection (0–4 doses) between those who developed long COVID and those who did not (*P* < .001). The great majority of vaccinated HCP (96.4%) received a heterologous COVID-19 vaccine scheme, and no difference was observed between cases and controls (*P* = .09). Overall, 887 HCP (12.6%) had 2 or more SARS-CoV-2 infections, including 17.8% of cases and 10.6% of controls (*P ≤* .001). Cases were less likely to be infected in the “omicron era” (24.7%) than controls (49.8%; *P* < .001). Interactions between COVID-19 vaccine doses prior to infection, number of SARS-CoV-2 infections, variant era and long COVID were not statistically significant (*P* > .05).

**Table 1.** Predictors of Long COVID^a^

Variable	Long COVID (N=1,993)	No Long COVID (N=5,118)	Unadjusted Analysis			Multivariable Analysis		
			OR	95% CI (ll–Ul)	*P* Value	OR	95% CI (ll–Ul)	*P* Value
Sex female	1,432 (74.1)	3,669 (71.7)	1.1	(1.00–1.27)	**.05**	**1.2**	**(1.05–1.39)**	**.008**
Age, mean y (SD)	38.1 (8.7)	37.2 (9.0)	1.0	(1.01–1.02)	**<.001**	**1.0**	**(1.00–1.02)**	**.003**
Body mass index	27.4 (4.9)	26.8 (4.8)	1.02	(1.01–1.03)	**<.001**	1.01	(1.00–1.03)	.096
Any comorbidity (N=5,722) present^b^	512 (31.1)	1,143 (28.0)	1.16	(1.02–1.31)	**.020**	1.05	(0.90–1.21)	.50
Hypertension	159 (9.7)	365 (9.0)	1.09	(0.89–1.32)	.397			
Diabetes	58 (3.5)	109 (2.7)	1.33	(0.96–1.83)	.084			
Arthritis	27 (1.6)	33 (0.8)	2.04	(1.22–3.41)	**.006**			
Stroke	1 (0.1)	12 (0.3)	0.21	(0.01–1.05)	.129			
Chronic kidney failure	3 (0.2)	8 (0.2)	0.93	(0.20–3.22)	.914			
Cancer	48 (2.9)	99 (2.4)	1.21	(0.85–1.70)	.290			
**Physical activity (N=5,722)** ^**b**^								
Never	554 (33.7)	1,454 (35.7)						
1 or 2 days/week	520 (31.6)	1,261 (30.9)	1.08	(0.94–1.25)	.273			
3 or 4 days/week	341 (20.7)	851 (20.9)	1.05	(0.90–1.23)	.535			
5 or 6 days/week	120 (7.3)	274 (6.7)	1.15	(0.91–1.45)	.247			
7 days/week	110 (6.7)	237 (5.8)	1.22	(0.95–1.55)	.116			
Direct patient-contact job	988 (51.1)	2,637 (51.5)	0.98	(0.89–1.09)	.758			
**Vaccine doses prior to infection**								
0	1,175 (60.8)	2,023 (39.5)						
1	96 (5.0)	184 (3.6)	0.90	(0.69–1.16)	.413	0.91	(0.60–1.39)	.677
2	272 (14.1)	665 (13.0)	0.70	(0.60–0.82)	**<.001**	1.17	(0.79–1.76)	.435
3	388 (20.1)	2,115 (41.3)	0.32	(0.28–0.36)	**<.001**	0.63	(0.39–1.02)	.058
4	2 (0.1)	131 (2.6)	0.03	(0.00–0.08)	**<.001**	**0.05**	**(0.01**–**0.19)**	**<.001**
Heterologous vaccine scheme administered(N=6,398)^c^	1,668 (95.7)	4,498 (96.6)	0.78	(0.59–1.03)	.077			
No. of SARS-CoV-2 infections								
1	1,588 (82.2)	4,576 (89.4)						
≥2	345 (17.8)	542 (10.6)	1.83	(1.58–2.12)	**<.001**	**1.27**	**(1.07**–**1.50)**	**.006**
**Variant era (N=6,918)** ^**d**^								
Non-VoC 2020	1,133 (59.8)	1,930 (38.4)						
Delta	43 (2.3)	214 (4.3)	0.34	(0.24–0.47)	**<.001**	**0.30**	**(0.17**–**0.50)**	**<.001**
Gamma	250 (13.2)	377 (7.5)	1.13	(0.95–1.35)	.174	1.03	(0.72–1.48)	.90
Omicron	469 (24.7)	2,502 (49.8)	0.32	(0.28–0.36)	**<.001**	**0.49**	**(0.30**–**0.78)**	**.003**

Note. VoC, variant of concern.

a Variables were included in multivariable analysis if significant univariate associations were shown. The 95% confidence intervals (CIs) of the odds ratios (ORs) have been adjusted for multiple testing. Independent predictors are shown in bold.

b Information available for 5,722 (81.1%).

c Any heterologous COVID-19 vaccine scheme.

d 113 healthcare personnel were not classified due to potential overlap between SARS-CoV-2 non-VoC and gamma, gamma and delta, and delta and omicron variants.

### Multivariable analysis

In the multivariable analysis assessing the association between exposure variables and long COVID, female sex (OR, 1.21; 95% CI, 1.05–1.39), older age (OR, 1.01 per year of age; 95% CI, 1.00–1.02), and 2 or more SARS-CoV infections (OR, 1.27; 95% CI, 1.07–1.50) were significantly associated with development of long COVID. Those infected with the SARS-CoV-2 δ (delta) variant (OR, 0.30; 95% CI, 0.17–0.50) or the o (omicron) variant (OR, 0.49; 95% CI, 0.30–0.78), and those who received 4 vaccine doses before infection (OR, 0.05; 95% CI, 0.01–0.19) were associated with a reduced risk of development of long COVID (Table 1 and Supplementary Appendix 4).

### Whole-genome sequencing analysis

During the study period, SARS-CoV-2 samples from 524 (6.6%) of the 7,979 RT-PCR positive test results were sequenced to determine the virus variant. Most of the samples were the SARS-CoV-2 δ (delta) variant (45.6%), followed by the o (omicron) variant (31.0%), and the γ (gamma) variant (20.8%). The SARS-CoV-2 variants α (alpha), B.1, B.1.1, B.1.1.28, and ζ (zeta) accounted for <1%, and 4 samples were inconclusive (0.8%) (Supplementary Appendix 5). The agreement between results of whole-genome sequencing analysis and the circulating variant eras that we defined was 438 (84.6%) of 518.

## Discussion

In this case–control study of HCP from Brazil, 27% had long COVID symptoms in the 6 months following infection. Acquiring >1 SARS-COV-2 infection was associated with a higher risk of long COVID. The most common long COVID symptom was headache, and about a half of those with long COVID had >2 symptoms. Risk factors associated with development of long COVID were female sex, age, and ≥2 SARS-CoV-2 infections. Protective factors were the SARS-CoV-2 δ (delta) variant, the SARS-CoV-2 o (omicron) variant, and receiving 4 doses of COVID-19 vaccine prior to infection.

A recent systematic review and meta-analysis demonstrated that long COVID is a public health issue, with an overall global estimated pooled prevalence of 43% (95% CI, 39%–46%), with a prevalence of 54% (95% CI, 44%–63%) in hospitalized patients and 34% (95% CI, 25%–46%) in nonhospitalized patients.^2^ Another systematic review including 57 studies reported that more than half of COVID-19 survivors experienced persistent post-COVID condition symptoms 6 months after recovery.^23^ Multiple papers have reported a variety of symptoms and durations to make a diagnosis of long COVID.^2,23–26^ The most common symptoms described in previous papers were fatigue or muscle weakness, persistent muscle pain, anxiety, memory problems, sleep problems, and shortness of breath.^2,23,25^ In our case–control study with up to 180 days of follow-up, headache, myalgia–arthralgia, nasal congestion, and fatigue were the most common symptoms. A previous paper from UK reported the major risk factors for not feeling fully recovered at 1 year after COVID-19 were female sex, obesity, and receiving invasive mechanical ventilation during the acute illness.^26^ Another study reported that regardless of the initial disease severity, COVID-19 survivors had longitudinal improvements in physical and mental health, with most returning to their original work within 2 years.^25^ In the multivariate analysis of our study, females were also more affected by long COVID than males. The reason for this is not clear but has also been shown in previous studies.^24,27,28^

Our study revealed that >1 COVID-19 infection was associated with long COVID among HCP. Previous evidence has shown that SARS-CoV-2 reinfection can increase the risk of having long-term health complications including all-cause mortality, hospitalization, and postacute sequelae.^10^ More attention should be given to COVID-19 among HCP, particularly when there’s a previous history of infection (ie, reinfection) and vaccination status.^10^

The SARS-CoV-2 VoC have multiple spike-protein mutations and appear to be more infectious or to cause more disease than other circulating variants.^29^ A growing body of early global research shows a clear effect of the vaccines against the new variants.^24,30–32^ Previous studies suggested that the SARS-CoV-2 δ (delta) and o (omicron) variants caused less systemic inflammatory processes, severe illness, or death, resulting in less severe long COVID symptoms than the wild-type variant (Wuhan).^33,34^ In addition, the prevalence of long COVID during the “omicron era” has been reported as less than that of the other strains, and a milder process during the acute phase might have been contributing to the less frequent development of long COVID during the “delta era” and the “omicron era.”^34,35^

The protective effect of COVID-19 vaccination versus long COVID is not well described. A recent systematic review and meta-analysis demonstrated that COVID-19 vaccination of at least 1 dose either before or after having COVID-19 significantly decreased the risk of long COVID-19.^36^ Recently, Azzolini et al^16^ demonstrated receiving 2 or more doses of COVID-19 vaccine before COVID-19 was associated with a lower prevalence of long COVID. However, in the present study, we revealed that receiving 4 doses of COVID-19 vaccinations before infection was a protective against long COVID, although a dose–response effect was not detected, which could have been due to the small sample size, creating an unbalanced comparison between vaccinated and unvaccinated. Vaccinations may play a role in preventing long COVID, and further studies need to be conducted with newer COVID-19 vaccinations included.

Our study had several limitations. First, we did not perform a test-negative case–control study because this study was retrospectively conducted using the data from symptom-based testing, and all included participants had documented COVID-19. Second, HCP may have had COVID-19, may have developed long COVID, but did not come back to the employee health clinic. If so, they would have been considered as not having long COVID, which whould have led to misclassification of the outcome. Third, since our institution offered COVID-19 testing for only symptomatic HCP, we did not include those with asymptomatic COVID-19, although long COVID can develop following asymptomatic infection.^1^ Fourth, a clearer and more standardized definition of long COVID is needed for researchers to investigate the true prevalence and to better evaluate the risk factors because there is no test to diagnose long COVID.^1,37^ Fifth, neutralizing viral antigen-binding antibody levels were not available in the vaccinated HCP in our study. However, the US FDA does not recommend antibody testing for SARS-CoV-2 to determine immunity or protection from COVID-19, especially among those who are vaccinated.^38^ Sixth, past medical history was missing for ∼20% of the study participants and those were excluded from the analysis. We also could not perform further analyses stratified by immunocompromised status due to the limited number of individuals (<3%) reported in our previous study.^39^ Because our study focused only on long COVID among HCP, we were not able to evaluate the impact of personal protective equipment. Finally, the most prevalent variant at the time of infection was used to assess long COVID risk because viral sequencing was limited to a small random sample of 524 HCP, corresponding to 7% of HCP diagnosed with symptomatic COVID-19 included in this study. However, the agreement between results of whole-genome sequencing analysis and the circulating variant eras that we defined was ∼85%.

In conclusion, our study demonstrated that long COVID can be prevalent among HCP. Importantly, multiple infections were associated with increased risk of long COVID, and the more recent SARS-CoV-2 δ (delta) and o (omicron) variants were associated with reduced risk. COVID-19 vaccines were significantly associated with reduced long COVID among HCP after additional doses (second booster). More studies are needed to evaluate long COVID after reinfection and additional genomic surveillance is required for a better understanding of vaccine effectiveness versus long COVID after infection with newer SARS-CoV-2 variants. The identification of risk and protective factors can facilitate the development of targeted prevention strategies for individuals with elevated risk of developing long COVID.

## Supplementary material

For supplementary material accompanying this paper visit https://doi.org/10.1017/ice.2023.95. click here to view supplementary material
